# *POGZ* de novo missense variants in neuropsychiatric disorders

**DOI:** 10.1002/mgg3.900

**Published:** 2019-07-25

**Authors:** Wenjing Zhao, Yingting Quan, Huidan Wu, Lin Han, Ting Bai, Linya Ma, Bin Li, Guanglei Xun, Jianjun Ou, Jingping Zhao, Zhengmao Hu, Hui Guo, Kun Xia

**Affiliations:** ^1^ Center of Medical Genetics & Hunan Key Laboratory of Medical Genetics, School of Life Sciences Central South University Changsha Hunan China; ^2^ The First People's Hospital of Yunnan Province Kunming Yunnan China; ^3^ National Clinical Research Center for Geriatric Disorders, Xiangya Hospital, Central South University Changsha Hunan China; ^4^ Mental Health Center of Shandong Province Jinan Shandong China; ^5^ Mental Health Institute of the Second Xiangya Hospital, Central South University Changsha Hunan China; ^6^ Hunan Key Laboratory of Animal Models for Human Diseases Changsha Hunan China; ^7^ Key Laboratory of Medical Information Research Central South University Changsha Hunan China; ^8^ CAS Center for Excellence in Brain Science and Intelligences Technology (CEBSIT) Shanghai China

**Keywords:** de novo, missense variants, neuropsychiatric disorders, *POGZ*

## Abstract

**Background:**

De novo likely gene‐disrupting variants of *POGZ* cause autism spectrum disorder (ASD) and intellectual disability. However, de novo missense variants of this gene were not well explored in neuropsychiatric disorders.

**Methods:**

The single‐molecule molecular inversion probes‐based targeted sequencing method was performed on the proband. Variant was validated using Sanger sequencing in both proband and parents. Immunoblot analysis was performed to examine the expression of *POGZ* in patient‐derived peripheral blood lymphocytes. Published *POGZ* de novo missense variants in neuropsychiatric disorders were reviewed.

**Results:**

We detected a novel de novo missense variant in *POGZ* (c.1534C>A, p.H512N, NM_015100.4) in an individual with ASD. Immunoblot analysis revealed a dramatic reduction in *POGZ* protein in patient‐derived peripheral blood lymphocytes suggesting a loss‐of‐function mechanism of this de novo missense variant. In addition, we collected and annotated additional eight *POGZ* de novo missense variants identified in neuropsychiatric disorders from literatures.

**Conclusion:**

Our findings will be beneficial to the functional analysis of POGZ in ASD pathogenesis, and for genetic counseling and clinical diagnosis of patients with *POGZ* de novo missense variants.

## 1 INTRODUCTION

Autism spectrum disorder (ASD) defines a group of neurodevelopmental disorder (NDD) characterized by impaired social communication, restricted interests and stereotyped and repetitive behaviors (First, 2013). Previous studies have shown that de novo variants, including copy number variation, and single nucleotide variants (SNVs) and small insertions and deletions (indels) were strongly associated with ASD patients (Chen, Penagarikano, Belgard, Swarup, & Geschwind, 2015; Sebat et al., 2007). Dozens of high‐risk ASD genes have been identified from the de novo perspective including *POGZ* (OMIM: 614787), which encodes a domesticated DNA transposase (Bartholomeeusen et al., 2009).

*POGZ* encodes a domesticated DNA transposase containing a cluster of multiple C_2_H_2_‐type ZNF domains, a HTH domain, and a DDE domain (Bartholomeeusen et al., 2009; Nozawa et al., 2010). The expression pattern of *POGZ* in fetal brain tissues suggests that POGZ may play an essential role in early embryonic development (Gudmundsdottir et al., 2018; Stessman et al., 2016). Previous studies have shown that POGZ is involved in neuronal proliferation, neurite outgrowth, chromatin remodeling, and gene transcription regulation (De Rubeis et al., 2014; Gudmundsdottir et al., 2018; Hashimoto et al., 2016; Nozawa et al., 2010). Genotype–phenotype correlation analysis has revealed that likely gene‐disrupting variants in *POGZ* define a potential ASD and ID syndrome (Stessman et al., 2016). However, the pathogenicity of de novo missense mutation of *POGZ* in ASD and other related NDDs is not clear. Here, we reported a *POGZ* de novo missense variant identified in an ASD patient. In addition, we comprehensively curated *POGZ* de novo missense variants from large‐scale sequencing studies of neuropsychiatric disorders and provided the evidence of their potential pathogenicity.

## 2 MATERIALS AND METHODS

### 2.1 Ethical compliance

The local institutional review board approved this study. Written informed consent was obtained from the family.

### 2.2 Patient presentation

**Figure 1:**
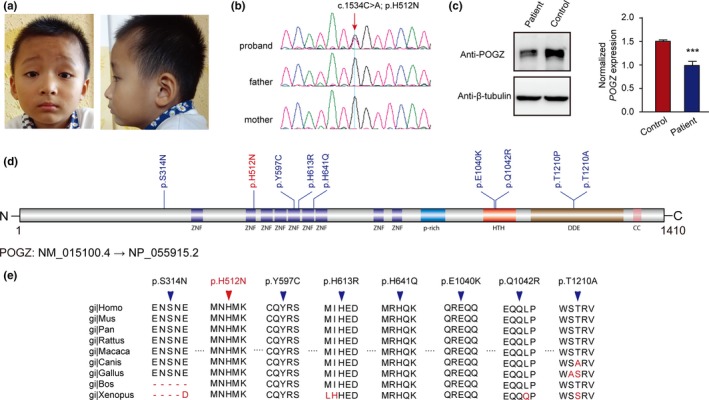
*POGZ* (GenBank accession number: NM_015100.4) de novo missense variants in neuropsychiatric disorders. (a) Frontal and lateral face photos of the proband reported in this study. (b) Sanger sequencing validated the missense variant is de novo. (c) Immunoblot analysis of *POGZ* expression in peripheral blood lymphocytes of patient and control. Three independent experiments were performed. Data are means ± *SEM*. Differences were statistically significant by Student's *t*‐test (***p < .001). (d) Location distribution of all reported *POGZ* de novo missense variants. The novel de novo missense variant identified in this study is marked with red color. (e) Conservation analysis of all reported *POGZ* de novo missense. The new pathogenic de novo missense variant identified in our study is denoted in red color

The male proband (Figure 1a) is the first child of his healthy, nonconsanguineous Chinese parents. He was born at term with weight of 3 kg and height of 50 cm. On examination at 5 years and 3 months of age, his weight was 20 kg, height 107 cm (−1 *SD*), and head circumference 52 cm. He was able to raise his head at the age of 3 months, sat without support at the age of 8 months, and walked without support at the age of 12 months. He has hypertonia and delayed fine motor coordination. Speech development was delayed. At the age of 10 months he started speaking his first words, at the age of 38 months he was able to speak his first phrases, at the age of 56 months he could speak complete sentences. His behaviors were characterized by mild attention problems, repetitive behaviors, rare stereotypies, communication difficulties, very little social interaction, temper tantrums, and poor eye contact. He was diagnosed as ASD according to the Diagnostic and Statistical Manual of Mental Disorders, 4th Edition (DSM‐IV) criteria and further confirmed by the Autism Diagnostic Interview‐Revised (ADI‐R). Medical concerns included strabismus in the left eye. There were no reported hyperactivity, gastrointestinal disturbances, sleep disturbances, aggressive behavior, and seizures. No significant facial dysmorphic feature was observed (Figure 1a). Brain magnetic resonance imaging performed at the age of 3 years was normal. G‐banded karyotyping revealed normal karyotype (46, XY).

### 2.3 Mutation detection and classification

Peripheral blood was collected from the proband and parents after obtaining written informed consent. Genomic DNA was extracted from the peripheral blood using a standard proteinase K digestion and phenol‐chloroform method. The single‐molecule molecular inversion probes (smMIP)‐based targeted sequencing method (Wang et al., 2016) was performed on the proband. *POGZ* (GenBank accession number: NM_015100.4) variant was validated using Sanger sequencing in both proband and parents.

### 2.4 Immunoblotting

EBV‐transformed lymphocyte cell lines from the patient and the controls were lysed in 2× SDS sample buffer (4% SDS, 20% glycerol, 10% 2‐mercaptoethanol, 0.004% bromphenol blue, 0.125 mol/L Tris HCl, pH 6.8) containing a cocktail of protease inhibitors (Millipore, Boston, MA, USA). Proteins were separated by SDS‐PAGE and transferred to polyvinylidene fluoride membranes. Membranes were incubated with anti‐human POGZ polyclonal antibo‐dies (NBP183004, Novus Biologicals, Littleton, CO, USA) overnight at 4°C. The membranes were incubated with secondary antibodies at room temperature for 1h. The signals were detected by using Immobilon Western Chemiluminescent HRP Substrate (Millipore).

## 3 RESULTS AND DISCUSSION

A rare heterozygous missense mutation in *POGZ* (c.1534C>A, p.H512N) was identified in a Chinese ASD patient with delayed speech and motor development (Figure 1a,b). Sanger sequencing validated this variant in the proband but not in the parents who verified that this variant is de novo. The variant is located in the predicted zinc finger (ZNF) domain, which is the most common DNA‐binding motif (Iuchi, 2001). The Combined Annotation Dependent Depletion (CADD) score is 28.9 and multiple in‐silico programs consistently predicted the deleterious effect (Table 1). To further confirm the pathogenesis of this mutation, we performed immunoblot using patient‐derived peripheral blood lymphocytes and revealed a drastic decrease in the POGZ (Figure 1c) suggesting a loss‐of‐function mechanism of this de novo missense mutation.

**Table 1:** *POGZ* de novo missense variants in neuropsychiatric disorders

Sample.ID	PMID	Mutation in gDNA (hg19,chr1)	Disorder	NTchange	AAchange	SIFT	Polyphen2	MutationTaster	CADD	ACMG classification
14483.p1	25363768	g.151400436C>T	ASD	c.941G>A	p.S314N	T	B	D	14.24	Likely pathogenic
SD0129.p1	this study	g.151396017G>T	ASD	c.1534C>A	p.H512N	D	D	D	28.9	Pathogenic
14551.p1	25363768	g.151384237T>C	ASD	c.1790A>G	p.Y597C	D	D	D	26.1	Likely pathogenic
1‐02312	26785492	g.151384189T>C	CHD/DD	c.1838A>G	p.H613R	D	D	D	25	Likely pathogenic
2‐1402‐003	28263302	g.151384104G>C	ASD	c.1923C>G	p.H641Q	D	D	D	27.5	Likely pathogenic
NA	25694107	g.151378393C>T	ASD	c.3118G>A	p.E1040K	D	D	D	31	Likely pathogenic
P1381	26582266	g.151378386T>C	ASD	c.3125A>G	p.Q1042R	D	D	D	24.8	Pathogenic
DDD4K.03715	28135719	g.151377883T>G	ID/DD	c.3628A>C	p.T1210P	D	B	N	11.9	Likely pathogenic
NIMH091221_Pro	23911319	g.151377883T>C	SCZ	c.3628A>G	p.T1210A	T	B	N	0.048	Uncertain significance

Note

*POGZ* GenBank accession number (NCBI Reference Sequence): NM_015100.4.

To explore the role of de novo missense variants of *POGZ* in neuropsychiatric disorders, we curated additional eight de novo missense variants (Table 1, Figure 1d) from seven large‐scale genome‐wide sequencing studies or individual patient reports (Deciphering Developmental Disorders Study, 2017; Fukai et al., 2015; Gulsuner et al., 2013; Hashimoto et al., 2016; Homsy et al., 2015; Iossifov et al., 2014; Yuen et al., 2017). De novo missense variants scatteredly located in the protein, no significant cluster was implicated. However, we observed two variants (p.T1210P, p.T1210A) at the same site and two variants (p.E1040K, p.Q1042R) in close proximity located in the DNA‐binding helix‐turn‐helix (HTH) domain. Most of the variants are conserved across species (Figure 1e) and predicted to be deleterious (Table 1). Notably, four de novo missense mutations are located in (p.H512N, p.Y597C and p.H641Q) or closed to (p.H613R) the predicted ZNF domains. We classified the variants following the standards and guidelines for the interpretation of sequence variants from the American College of Medical Genetics and Genomics (ACMG) (Richards et al., 2015) and provided the classification information in Table 1. In summary, we identified a novel de novo *POGZ* mutation in an ASD patient and interpreted the potential pathogenicity of de novo missense variants. Our findings will not only benefit the clinical diagnosis and genetic counseling but also provide pathogenic missense variant for the study of POGZ‐related pathogenesis.

## CONFLICT OF INTEREST

The authors declare no conflict of interest.
